# Newborn Screening for Cystic Fibrosis: A Qualitative Study of Successes and Challenges from Universal Screening in the United States

**DOI:** 10.3390/ijns8030038

**Published:** 2022-06-23

**Authors:** Marci K. Sontag, Joshua I. Miller, Sarah McKasson, Amy Gaviglio, Stacey L. Martiniano, Rhonda West, Marisol Vazquez, Clement L. Ren, Philip M. Farrell, Susanna A. McColley, Yvonne Kellar-Guenther

**Affiliations:** 1 Center for Public Health Innovation at CI International, Littleton, CO 80120, USA; jmiller@ciinternational.com (J.I.M.); smckasson@ciinternational.com (S.M.); rwest@ciinternational.com (R.W.); ykellar-guenther@ciinternational.com (Y.K.-G.); 2 Colorado School of Public Health, University of Colorado Anschutz Medical Center, Aurora, CO 80045, USA; 3 Connetics Consulting, Minneapolis, MN 55417, USA; amy.gaviglio@outlook.com; 4 Children’s Hospital Colorado, Aurora, CO 80045, USA; stacey.martiniano@childrenscolorado.org; 5 Department of Pediatrics, University of Colorado School of Medicine, Aurora, CO 80045, USA; 6 Ann & Robert H. Lurie Children’s Hospital, Chicago, IL 60611, USA; MarVazquez@luriechildrens.org (M.V.); smccolley@luriechildrens.org (S.A.M.); 7 Department of Pediatrics, Division of Pulmonary and Sleep Medicine, Northwestern University Feinberg School of Medicine, Chicago, IL 60611, USA; 8 Children’s Hospital of Philadelphia, Philadelphia, PA 19104, USA; renc@chop.edu; 9 Division of Pulmonary and Sleep Medicine, University of Pennsylvania Perelman School of Medicine, Philadelphia, PA 19104, USA; 10 Departments of Pediatrics and Population Health Sciences, University of Wisconsin-Madison School of Medicine and Public Health, Madison, WI 53705, USA; pmfarrell@wisc.edu

**Keywords:** cystic fibrosis, newborn screening, diagnosis, primary care providers, timeliness, qualitative

## Abstract

Cystic fibrosis (CF) newborn screening (NBS) was universally adopted in 2009 in the United States. Variations in NBS practices between states may impact the timing of diagnosis and intervention. Quantitative metrics can provide insight into NBS programs (NBSP), but the nuances cannot be elucidated without additional feedback from programs. This study was designed to determine facilitators and barriers to timely diagnosis and intervention following NBS for CF. The median age at the first CF event for infants with CF within each state was used to define early and late states (*n* = 15 per group); multiple CF centers were invited in states with more than two CF centers. Thirty states were eligible, and 61 NBSP and CF centers were invited to participate in structured interviews to determine facilitators and barriers. Once saturation of themes was reached, no other interviews were conducted. Forty-five interviews were conducted (*n* = 16 early CF center, *n* = 12 late CF center, *n* = 11 early NBSP, and *n* = 6 late NBSP). Most interviewees reported good communication between CF centers and NBSP. Communication between primary care providers (PCPs) and families was identified as a challenge, leading to delays in referral and subsequent diagnosis. The misperception of low clinical risk in infants from racial and ethnic minority groups was a barrier to early diagnostic evaluation for all groups. NBSP and CF centers have strong relationships. Early diagnosis may be facilitated through more engagement with PCPs. Quality improvement initiatives should focus on continuing strong partnerships between CF centers and NBS programs, improving education, communication strategies, and partnerships with PCPs, and improving CF NBS timeliness and accuracy.

## 1. Introduction

Cystic fibrosis (CF) newborn screening (NBS) was introduced in the United States (US) in the 1980s, followed by slow but steady adoption of CF screening through the 1990s. Universal NBS for CF was recommended in a pivotal report in CDC’s Morbidity and Mortality Weekly 2004 Report (MMWR), leading to further expansion of CF newborn screening throughout the US. By late 2009, all 50 states and the District of Columbia had implemented CF NBS.

While all states now screen for CF, there are variations in the approach. Collection and transport of dried blood spot specimens, screening algorithms, communication of results to stakeholders, and education of stakeholders vary between states. All CF NBS algorithms first measure immunoreactive trypsinogen (IRT), a pancreatic enzyme precursor associated with pancreatic inflammation, albeit not a specific biomarker for CF. Variation occurs in whether and when a second assessment of IRT occurs, when and whether a second screening specimen is required, which may be in the first few days or weeks of life, and whether a fixed or floating cutoff value is used. When IRT is out of range according to program criteria, a cystic fibrosis transmembrane conductance regulator (*CFTR)* gene variant panel from the first or second NBS dried blood spot specimen [1,2,3] is performed. Some states add full *CFTR* gene sequencing when only one variant is identified [4]. Evaluation of sensitivity for identifying infants with CF has prompted programs to lower IRT cutoffs, apply floating cutoffs, and implement different *CFTR* variant testing approaches [5,6,7]. In addition to IRT cutoffs, birth census, geography, demographics, accessibility of diagnostic facilities, use of prenatal testing, parent and primary care provider (PCP) knowledge of CF and NBS, communication strategies around a positive CF NBS, and how diagnostic testing is scheduled also vary between states. These factors may influence the outcomes of an infant who has a positive CF NBS.

We previously described the outcomes of infants born with CF since the universal implementation of NBS in the US [8]. We found variation in the age at the first CF-related event (sweat test, clinical encounter, or care episode) between infants. This study sought to understand programmatic differences that may be facilitators or barriers to timely and accurate CF diagnosis and initial treatment for US infants with CF. We interviewed US CF centers and state NBS programs to identify themes that influenced variation and may drive improvement in CF NBS.

## 2. Materials and Methods

We used the median age at first CF event (AFE) data from the 2018 CF Foundation Patient Registry (CFFPR) to identify the 15 states with the earliest median AFE (early) and the 15 states with the latest AFE (late). The AFE composite measure is based on the earliest date of the first sweat test, clinical encounter, and/or hospitalization [8]. We invited NBS personnel from state laboratories and follow-up programs to participate in interviews. We also invited personnel from one to three CF centers in each identified state to participate, according to the number of CF centers in the state. Invitations were sent via email to NBS programs and CF center pediatric program directors, with up to three reminder emails sent to nonrespondents. To investigate the demographic similarities between the early and late groups, we extracted the percent of low birthweight and premature babies born in each state from the National Center for Health Statistics [9].

We developed an interview script informed by polls (1) of attendees at the annual Cystic Fibrosis Foundation (CFF) Newborn Screening Quality Improvement Consortium meeting in 2019, and (2) State CF NBS Program participants in a national webinar in 2019. Participants at these two events responded to a series of six multiple-choice, free-response, and ranking questions in Poll Everywhere™ designed to understand the barriers and facilitators in CF NBS and the opportunities for maximizing outcomes. The interview script was piloted with CF centers and NBS programs from two states. Interviews often included multiple participants from the same program type (i.e., CF center or state NBS program) and were conducted over Zoom™ using scripted guides. Interviews were recorded for transcription purposes, and transcription was completed through the commercially available system Rev (www.rev.com, last accessed 1 November 2020). Participants were offered a coffee shop gift card for participating.

Interview transcripts were coded in Nvivo v.11 by one of three coders using the constant comparative technique; 14 were dual coded with 94% to 100% agreement. To determine when saturation was reached, the researchers utilized Guest, Namey, and Chen’s (2020) approach, looking at the percentage of new scores identified and their suggested target of less than 5% of the total codes identified when coding the last few interviews [10].

Themes are reported when at least 30% of either group mentioned the theme. The frequency of the reported theme in each group is presented in tabular form in two categories: at least 60% of the interviews reporting the theme, or 30–60% of the interviews reporting the theme. If a theme was not mentioned in an interview for a given group or mentioned by fewer than 30% of respondents, the corresponding table cell is empty. In addition, representative quotes from participants are presented to illuminate the themes identified. The term “variant” is used throughout the manuscript to refer to *CFTR* gene variations; however, the historic nomenclature is maintained in quotes from interviews that mention “mutations”.

## 3. Results

### 3.1. Participants

Thirty states were identified as potential participants (79 total organizations: 30 state NBS programs, 49 CF centers). The median AFE of the 15 early states was 18 days, compared to 28.5 days in the 15 late states. Invitations were sent in a staged approach with approximately eight states in each stage; saturation was tested after each stage. Through an iterative process and to assess saturation, the coders evaluated new codes as additional interview data were evaluated. We reached a saturation ratio of 0.06 for the NBS programs and 0.04 for the CF centers after 45 interviews. In total, 22 NBS programs were invited, and 17 participated; 40 CF centers were invited, and 28 participated. Specifically, 11 early NBS programs, 16 early CF centers, six late NBS programs, and 12 late CF centers were interviewed. Not all identified CF centers and NBS programs were invited to participate in the interviews because the saturation of themes was reached. Representation from all regions in the US was confirmed (Southwest 50%, Southeast 25%, Northeast 55%, Midwest 50%, West 36%) [11].

When looking at National Health Statistics Data [9], there were no differences in the early vs. late states in the frequency of low-birthweight babies (2020 Data, Category A: 6.5 < 7.56%, B: 7.56 <.62%, C 8.62 < 9.68%, D: 9.68 < 10.74%, E, 10.74 < 11.8%); early: Category A: 3/13 (23%), B: 6/13 (46%), C: 4/13 (31%), D: 0/13, E: 0/13; late: A: 3/8 (37.5%), B: 2/8 (25%), C: 2/8 (25%).D: 0/8 (0%), E: 1/8 (12.5%)) or preterm birth rates (2020 Data, Category A: 7.62 < 8.93%, B: 8.93 < 10.25%, C 10.25 < 11.56%, D: 11.56 < 12.88%, E, 12.88 < 14.19%); early: Category A: 2/13 (15%), B: 6/13 (46%), C: 3/13 (23%), D: 1/13 (8%), E: 0/13 (0%); late: A: 1/8 (12.5%), B: 3/8 (37.5%), C: 3/8 (37.5%).D: 0/8 (0%), E: 1/8 (12.5%)).

### 3.2. Facilitators to Timely CF Confirmatory Diagnosis and Care

#### 3.2.1. NBS Program and CF Center Communication

The most consistent facilitator for timely CF diagnosis and treatment was the strong relationship and communication between the NBS programs and CF centers (Table 1). NBS programs felt the CF centers were good about reporting back diagnostic outcomes and false negative (or missed) cases. In addition, both CF centers and NBS programs felt the collaboration between all parts of the CF NBS system worked well to make sure families were notified about a high-risk or presumptive positive CF NBS result.

“*I think the communication works well both ways, too. So, for example, if we have a sibling of one of our patients that’s born, so we know that there’s already a 25% chance, we then have the ability to sort of reach out directly to that contact person [at state NBS lab] to say, ‘A sibling’s been born, we need to run genetic testing sort of immediately.’ So, I feel like the communication goes back and forth pretty easily between our team and [state NBS program].*”

(Late CF center)

“*I think just to specifically call out the strengths of the communication between the newborn screening program and our center with the very high-risk infants. I can’t think of a single instance in the last couple of years where a child who we were notified about having two known disease-causing mutations was not rapidly referred if the pediatrician or the family expressed an interest to come to our center.*”

(Early CF center)

Interviewees reported their state CF centers and NBS programs had formal meetings ranging from biweekly to annually, with the majority meeting one to two times a year. Respondents also reported that they would informally meet to address a specific identified issue if the need arose. All but two CF center interviews stated there was strong communication between their CF center and the NBS program.

“*Outside of [regular] newborn screening, when issues arise, we may receive an email from the director of the newborn screening program… [for example] we are transitioning over to a new test because the previous one was recalled. We had emergency meetings for those. [The NBS Director] was really quick about informing and getting everybody on a meeting quickly so that we could resolve the issue and put a plan in place that was agreeable to all centers in our state*.”

(Early CF center)

When asked about communication around referrals from infants in adjacent states, only three interviewees reported a lack of good communication with their neighboring states, while 13 felt it was strong. NBS programs reported communicating with CF centers, PCPs, and NBS programs in other states to get needed information for babies whose care had crossed state lines. CF centers reported that they could communicate with neighboring state CF centers and NBS programs. While a higher percentage of the early CF centers and NBS programs stated they had good communication with neighboring state programs, all four groups mentioned this theme.

#### 3.2.2. Streamlined Scheduling and Diagnostic Testing Process

The final facilitator identified through the interviews was a streamlined sweat testing process. Respondents mentioned that their sweat testing process was smooth because of their approach to scheduling, notifying all partners of screening results, scheduling diagnostic testing, and getting families into care quickly.

“*I feel like our process, once we have the baby in our queue, goes really smoothly. They get scheduled; we follow up if they don’t come to their appointment if they’re intermediate. We have good processes in place once they get to our institution. I think that works well, and we have a good, closed loop with pediatricians to say, ‘Hey, we saw this baby, and this is what we found out.’ We send a letter that day to the pediatrician or call them if it’s a positive kid. I think once they enter the hospital, it works well*.”

(Early CF center)

*“I think that their approach to dealing with these babies is that when they’re first seen, they have them scheduled with a particular physician, do the sweat test, see the physician, and get the sweat test results all together. It’s all in one day, so they’re going to walk out of there with at least a partial answer because sometimes you get the equivocal sweat test. At least then they have a plan. But it’s not like they go there, have the sweat test and wait for 3 days for the doctor to call them*.”

(Early NBS program)

“*I think the centers are good about reporting [diagnostic results] to us. I just think sometimes it takes longer for CF than our other disorders to get a diagnosis, to get that information back*.”

(Late NBS program)

“*And actually, I feel like a big family. Everybody knows everybody. So, one way or another, that information will come*.”

(Early NBS program)

### 3.3. Barriers to Timely CF Confirmatory Diagnosis and Care

#### 3.3.1. Initial Referral and Risk Communication as a Barrier

When asked about barriers to timely CF confirmatory diagnostic testing and care, the primary barrier identified was the referral process. (Table 2) PCPs are usually and often solely responsible for notifying parents and referring infants to a CF center. This process is based on the expertise that healthcare providers have in delivering test results, and PCPs may also have an established relationship with the family through a prenatal visit or their care of a sibling. However, the referral process can be a rate-limiting step in some cases.

“*Well, the hard part at least is that [CF center] can’t really call them until we know the pediatrician has spoken to them. Because, otherwise, it’s pretty upsetting that they don’t hear it [positive CF NBS screen result] from somebody they know*.”

(Late CF center)

How the PCP notifies the family is essential. Many interviewees felt PCPs did a great job overall.

*“… My experience over the years, almost without exception, is that the PCP doesn’t say, ‘The newborn screening results say that your child has CF.’ They will say, ‘The newborn screening results show that there’s an abnormal result, so we’re referring you to the CF Center*.’”

(Early CF center)

However, some interviewees identified opportunities for improvement with PCPs. Fifteen interviewees felt that the process would be improved by better-informed risk communication from PCPs to families and a better understanding of the screening algorithm used in the state. These were common themes in the late CF center and late NBS program groups (Table 2).

*“… Sometimes, the pediatricians will tell the families not to come in for the evaluation, and instead, they’ll just repeat the IRT somewhere down the road. So sometimes we don’t see people, and they’ve had three IRT specimens repeated, and then finally they’ll come to see us, and it’s positive*.”

(Late CF center)

“*We still have a lot of small-town family doctors… who still think of CF as a death sentence. Some of our patients, they have been told that by their PCP*.”

(Early CF center)

PCPs delaying the referral and poor communication with the families were noted as barriers by the late CF center and NBS program groups, but not by the early groups. In addition, respondents indicated that sometimes the PCP might delay referral to CF centers for a sweat test because they believe that only White non-Hispanic people are at risk for CF.

“*We have several infants in a particular area that are lost to follow up. After the third or fourth IRT is still elevated, but the [case] was closed, lost to follow up. Could that have been a CF case? Who knows? I feel like they don’t take [the algorithm] seriously like maybe we’re crying wolf or something… It’s an area of the country where it’s predominantly African American, so I think there may be a misconception that CF can’t happen in this particular population. Therefore, they’re not aggressive in pursuing follow-up with that infant*.”

(Late CF center)

“*A lot of pediatricians, especially in [part of state], don’t believe Hispanic patients can have CF. So, it may be if only one mutation is picked up, they may drag their feet about referring the kid because of this misconception*.”

(Late CF center)

When asked what they wish PCPs knew, participants felt they should receive education on (1) NBS processes, including how to read the results, (2) how to communicate the appropriate risk of CF after NBS to families, and (3) the importance of quickly notifying families and getting them connected to a CF center. (Table 3)

“*For them to know what treatment and what getting earlier treatment means to the child, and just digestive enzymes to start, so you don’t have the failure to thrive, so you don’t have those other issues, I think that there are a lot of PCPs that don’t understand that, that it’s not just we’re worried about the baby’s breathing*.”

(Late NBS program)

PCPs may be misled about the importance of timeliness since the education materials may not stress the time-sensitive nature of CF diagnostic testing. As a result, another suggestion around PCP education was that the materials given to them with a positive NBS might need to be edited to highlight the importance of quickly connecting families to a CF center.

“*The materials that are used that are sent to the PCP from the state are very good materials. A lot of it’s from the CF Foundation, but I don’t feel a sense of urgency in those materials.*”

(Early CF center).

#### 3.3.2. Infant and Family Barriers

Participants identified two key themes related to the infant and family as barriers to timely sweat testing (Table 4). The first was a delay in sweat testing due to the infant being too young, being too small, or being in the NICU. This was only mentioned in the early CF and NBS groups.

“*The earlier we do it, as you know, the more likelihood that there’s going to be a QNS result. On some occasions, we have tried to do it earlier like two weeks of age or younger, sometimes we do get a QNS result. When we’re attempting the sweat test at that age, we’re usually just upfront with their family that there’s a possibility that we might not be able to collect enough sweat, but if we do collect enough sweat, then we can get an answer*.”

(Early CF center)

The second barrier was locating the family, making contact with them, and scheduling a sweat test.

*“… It translates to the parent’s priority to get it done, depending on what they perceive is the impact of it… We had to call [child protective services]… get the juvenile officer involved and everything, just to get them in for the sweat test to let them know your baby has CF, and this is what we’ve got to do*.”

(Early CF)

#### 3.3.3. Racial, Ethnic, and Geographical Barriers

When asked about barriers to timely sweat testing for infants from racial and ethnic minority groups, the most common theme identified was families lacking resources due to their socioeconomic status (Table 5).

“*I think the disparity more in lies with socioeconomic status than it does in minority grouping or ethnicity*”

(Early NBS program).

In addition, the ability to take time off work to complete the sweat test process, especially when significant travel or transportation needs were required, was mentioned.

“*And then also, just having the lack of sweat testing capability throughout the state… if you live in the Southern part of the state, you have to come all the way up to [city area]. It is not a test that is easily run after hours. So, if you have a job… it is harder to get into and make an appointment*.”

(Late NBS Program).

Only the early CF center and NBS program groups identified missing potential CF cases due to a limited variant panel as a potential barrier (*n* = 12 interviewees). The specific language from the interviews varied (variant panel not big enough, variant panel does not include variants from minority groups, etc.) and were categorized into this theme. Additional reasons mentioned by 12 interviewees were communication barriers (language barrier, need for a cultural broker, and low education level) and geographic barriers to traveling to the sweat test locations. Families driving long distances for sweat testing and not showing up for the testing were the most frequently mentioned delays to sweat testing, regardless of racial and ethnic background.

The third most frequently mentioned racial barrier was the myth that “only White people can have CF”, a concern for both the PCPs and the families.

“*Some of the barriers we still experience are kind of beliefs in the community that only [White] people can have CF, and so sometimes we have delays in diagnosis because even if you have a positive newborn screen, the family is given information like, ‘Oh, you probably don’t have it because you’re not white.’ And so, then the family doesn’t come to seek help.”*

(Late NBS program).

“*We had a baby that was African American last year that the parents were like, ‘Well, there’s no way that our baby has CF because that’s a white people’s disease.’ It ended up being disastrous for the baby*.”

(Early CF Center)

### 3.4. Themes Identified within the Screening System

#### 3.4.1. CF NBS Algorithms

NBS programs and some CF centers felt the IRT cutoffs for their state were appropriate for minimizing false positive and negative results. Nine interviewees stated they had a reasonable cutoff, and 11 said they did not believe they were missing many CF cases in their state. The early CF center group most commonly felt the IRT/DNA screening model worked, while the late NBS program group felt the IRT/IRT/DNA worked well because it was cost-effective.

“*I think it’s a good thing, the IRT/IRT/DNA algorithm… does reduce false positives a lot and reduces identification of carriers. It reduces the number of CF DNA we have to perform*.”

(Late NBS program)

“*Well, they use a floating IRT cutoff, several thousand specimens that run each day, and it’s the top 5%… we’ve been doing this for, well, the full gene sequencing portion of it for two and a half years now. And I don’t know of any missed cases…*”

(Early CF center)

While 12 interviewees, primarily Late CF centers, felt the *CFTR* variant panel worked for the population in their state, over one-third of the early CF Center group felt the panel used in their state was not large enough. In addition, this group was worried that they were missing infants from racial and ethnic minority groups who might have CF.

“*I think what works well too is that our panel is fine-tuned to capture all mutations by race and ethnicity. It wasn’t just looking at populations like White European that are known to have higher prevalence to CF; it was incorporating Hispanics and African Americans and their mutation frequencies into the panel*.”

(Late NBS program)

*“[We need to have] expanded the molecular testing at the department of health newborn screening level. Then, hopefully… a rarer variant like that [discussed earlier] would be intercepted at birth*”

(Early CF center).

#### 3.4.2. Difference in Notification and Referral Practices Based on Number of Variants Identified

All groups noted a more urgent notification from the NBS program dependent upon the number of *CFTR* gene variants detected in the NBS (Table 6). For example, the NBS programs and CF centers acted faster on a positive NBS that showed two *CFTR* variants rather than just one variant. In addition, all groups noted that PCPs and specialists were notified more quickly for results with two variants.

For results with two variants, interviewees clarified that not only did the call happen more quickly, but they notified more people initially. Of the 17 interviewees that discussed this theme, seven stated they notified the PCP (41%), four (24%) stated they notified the CF center/pulmonologist, and six (35%) indicated they notified multiple groups at once. However, of the 14 interviewees that mentioned personnel notified for one *CFTR* variant, eight said the PCP only (57%), two said the CF center (14%), and four said the PCP and CF center (29%).

“*If it’s… two mutations, the doctor, the pulmonologist on call is paged by the laboratory. They are given the information, they notify the CF coordinator, and they call the pediatrician on call*.”

(Early CF center)

Five interviewees also noted that the message relayed to the family also differs when the NBS result identifies two *CFTR* variants versus one variant. For example, for one-variant results, the early CF center group noted that the message to the family might be that the child is likely a carrier. The early CF center group also stated less urgency in notifying the family of the positive NBS and scheduling confirmatory testing when only one variant was detected. However, just as with notification, respondents reported more urgency for getting newborns with two identified CF variants in for treatment and diagnostic testing.

“*If there are two mutations… I’m going to get the sweat test. I’ll see them whenever they want to be seen that day… Because if two mutations… you got to feel reasonably comfortable that this will probably be cystic fibrosis, even though it’s not diagnostic*.”

(Early CF center)

“*Now if it’s a two-gene (variant) baby that’s coming in… A lot of times, we don’t do the sweat test until we see that the baby is nutritionally doing well… If we could give them enzymes and salt and get the sweat by a month and be fine, and that’s generally how we do that*.”

(Late CF center)

#### 3.4.3. Difference in Utilization of Genetic Counselors

We asked all interviewees if they utilized genetic counselors. Of the 45 interviewees, 11 (24%) reported that a genetic counselor with the NBS program was involved in CF NBS, and 27 (60%) said a genetic counselor from the CF center was involved. Stratifying by analytic group, over 80% of both the early CF (13/16) and the early NBS (9/11) groups reported that there were genetic counselors in the CF center. Only one late NBS program and four late CF centers stated they had a genetic counselor in the CF center. The presence of genetic counselors in the CF centers was the only theme reported more frequently by both early groups.

The genetic counselors provided education on carrier status and family planning. In some programs, all families see a genetic counselor; however, for many, the genetic counselor talks to families with one mutation.

“*I also like the fact that we have the genetic counseling available, so even if you are a carrier, you go for the genetic counseling. And I feel like it’s great because their parents understand what the genetic counseling means for future pregnancies. And their family gets a typewritten letter that they can save for the child and goes into their medical record. And they have that for a lifetime*.”

(Early CF center)

## 4. Discussion

There were few differences in practice reported between the early and late groups. Respondents in both early and late groups identified strong communication between NBS programs and CF centers as the primary facilitator for timely NBS and sweat testing. Both groups also identified similar challenges, including racial and ethnic disparities, delayed referrals for sweat testing, and programmatic challenges within the NBS system [12]. Nevertheless, identified themes point to opportunities for systematic changes in efforts to improve the quality and timeliness of CF NBS. For example, a higher percentage of the late NBS programs and CF centers identified delayed referral by the PCP, geographic distance to sweat testing centers, and the time commitment for completing sweat testing as obstacles. The early NBS programs and CF centers, on the other hand, were more likely to note transportation barriers and the potential for missed cases due to the limited number of *CFTR* variants on NBS panels as barriers.

This study highlights a heightened need to improve education and communication with PCPs. Late CF centers and NBS programs both noted delayed referral and communication from PCPs regarding positive NBS results. This was one of the few themes where we observed differences between the early and late groups, highlighting a key potential contributor to diagnostic testing and intervention delays. PCPs are usually the first recipients of an out-of-range NBS result and are responsible for notifying the family and making the appropriate, timely referrals. The perceived lack of knowledge among PCPs around the importance of quickly notifying the family of a positive screen was a common barrier identified. CF centers noted that PCPs did not always have updated information about CF to convey the risk of CF adequately or the need for timely sweat testing to families, and NBS programs more often identified PCPs as not recognizing the importance of sharing the positive NBS results in a timely manner with the appropriate level of urgency. PCP uncertainty in managing positive NBS results [13] and challenges facing parents in getting access to appropriate care following NBS [14] were described and are opportunities for improvement. We identified the gaps in the system for PCP knowledge and communication through subjective interviews with CF center and NBS program staff rather than a systematic assessment of PCP knowledge. A bias was likely introduced such that the interviewees were likely to highlight the challenging cases that rose to the attention of the CF centers and NBS programs. While seamless transitions through diagnosis and into clinical care are likely frequent, the interviewer prompts sought opportunities for improvement (e.g., “What do you wish PCPs knew about the newborn screening system?”). Furthermore, the interviews did not include representatives from the PCP community, hindering our ability to elucidate the PCP view on limitations within the system. These observations offer opportunities for improved communication and education among all stakeholders, confirming recent findings that called for additional education of PCPs on genetic disorders, test results, and counseling families in the context of CF [15].

Providing better education and communication strategies for PCPs is essential for the NBS and CF community. NBS programs and CF centers are responsible for ensuring that high-quality, informative communication is delivered at the time of notification. The communication should include the risk of having CF after a positive NBS result, updated CF clinical information, and appropriate gravitas to encourage the family to act upon the recommendations. Three specific opportunities for education are as follows: (1) the misunderstanding that a positive CF NBS with a single pathogenic *CFTR* variant is associated with a low risk for CF, while the actual risk varies considerably on the basis of the CF NBS algorithm and genetic heterogeneity of the population; (2) significant morbidity and mortality can occur with delays in CF diagnosis, including hyponatremic dehydration, impaired growth or severe malnutrition, and irreversible lung disease (bronchiectasis) [16,17]; (3) the refuted dogma that adequate sweat volumes cannot be collected until 2–3 months of age [18,19]. While not explicitly addressed in this study, communication and education systems among the early states may provide opportunities for benchmarking and quality improvement goals.

Although not identified as a barrier, we found significant variability between participating states in algorithms, communication, and follow-up after a positive NBS. Early programs identified limited NBS *CFTR* variant panels as the primary barrier to timely diagnosis. Late programs did not identify *CFTR* variant panels as barriers, although this may have been recognized but not mentioned. National standards exist for CF NBS [20], diagnosis of CF following NBS [21], the care of infants diagnosed by NBS [22], and recommendations for timeliness for the overall NBS system [12]. Clinical and Laboratory Standards Institute (CLSI) guidelines also outline procedures for determining the appropriate algorithm, cutoffs, *CFTR* variant panel for CF NBS programs, and follow-up processes but do not recommend specific practices. Ultimately, NBS is managed by state public health departments that have the autonomy to determine methods, algorithms, and IRT cutoffs. The implementation of varying approaches may result in delayed diagnosis and treatment. In addition, the timeliness of result reporting, follow-up, and sweat test may be impacted by the number of *CFTR* variants identified on the NBS panel. Specifically, the urgency of follow-up and sweat tests in infants with only one variant identified points to differences in approach in early CF centers. Interviewees reported more urgency for those newborns with two variants compared to one, with an impact seen on the timing and recommendations to schedule diagnostic testing and start treatment. The exception was the early CF centers, which did not note a difference in timing and recommendations for positive results with one variant vs. two variants. Since all other participant groups stated this, this likely contributes to early AFE. While we cannot establish cause and effect in this qualitative study, the patterns of the early CF groups warrant further investigation. They may be relevant to quality improvement efforts to improve the timeliness of AFE. A specific outcome of higher urgency for two variants compared to one variant is that there may be a delay in diagnosis for infants who are demographically from racial and ethnic minority groups, and who may be more likely to have *CFTR* variants that are not be included on the NBS *CFTR* variant panel [23].

One critical theme across all groups is the misconception that infants from racial and ethnic minority groups are at low risk for CF. Despite expanding literature on health disparities experienced by racial and ethnic minority groups with CF [7,24,25,26,27,28,29], the misconception of CF as a disorder occurring primarily in the non-Hispanic White population persists. When these disparities occur after diagnosis, they result in poor clinical outcomes. The current study provides evidence that the initial evaluation and diagnosis following a positive NBS may be delayed due to the mistaken belief that infants from racial and ethnic minority groups will not have CF. The theme was identified in both the early and late groups, highlighting the need for continued education and outreach to PCPs and others responsible for relaying the NBS results to families. Early CF centers and NBS programs were also more likely to identify the need for a larger *CFTR* variant panel in their state’s NBS laboratory to target *CFTR* variants in minority patients [8,30]. While each of these states may have a panel that has fewer variants, one could also postulate that the early interviewees are more knowledgeable about the NBS algorithm decisions in their states. The awareness of the algorithms and active engagement in decision making might be a component of what puts those programs in the early group. These were not the only barriers to timely diagnosis within minority groups. The other systemic barriers that were mentioned include socioeconomic status, geographic distance, transportation challenges, and the time commitment required for diagnostic testing. Implicit bias in healthcare is common [31], and continued efforts need to be employed to eliminate diagnostic biases in CF, beginning with NBS communication and referral, and extending into societal systems that may disproportionately impact minority groups.

While we designed this study to identify differences between states with early and late AFE, the lack of differences in responses could be because the relationships between the NBS programs and CF centers that took part in the study were strong. The primary facilitator highlighted for timely CF diagnosis was strong communication. Only one program stated that the relationship was problematic. The finding that there are strong relationships and good communication between CF centers and NBS programs indicates that a collaborative approach to improving processes, including communication with PCPs, is feasible and may also be associated with earlier intervention for newborns.

This qualitative assessment of CF NBS had limitations. First, the number of states interviewed was small, and some invited programs did not choose to participate in the interviews. There could be a self-selection bias as more early states participated than late states. Those who chose to participate may have better relationships between the NBS programs and CF centers, which could have diminished the observed differences. The interviews were conducted during the public health COVID-19 response; programs that could not participate may have been less resourced.

Furthermore, we defined the early and late states on the basis of 2018 CFFPR data. AFE from the CFFPR has been described elsewhere [8] and appears to be associated with longer-term outcomes. However, different states may have been selected for interviews had different metrics been chosen, such as age at diagnosis or the time from specimen collection to the receipt of specimen at the laboratory from NBS programs [32]. This variability could have influenced the stratification of early and late states and ultimately affected our conclusions. Lastly, not every interviewee was asked every question due to time constraints. However, they were all asked to describe their process and recommendations for other groups.

## 5. Conclusions

CF NBS has been successfully implemented in the US, yet there are many opportunities for improvement. Some improvement opportunities are unique to CF, while others offer insights into the broader NBS system. We are committed to continuing to support the broader CF newborn screening system through quality improvement efforts focused on continuing strong partnerships between CF centers and NBS programs, improving education, communication strategies, and partnerships with PCPs, and improving CF NBS timeliness and accuracy. We are also seeking opportunities to support ongoing work to reduce racial and ethnic disparities in NBS. Communication and educational strategies for CF can be applied broadly to other NBS disorders, improving the quality of the entire NBS system and ultimately improving outcomes for infants at risk for disorders identified by NBS.

## Figures and Tables

**Table 1:** Key themes identified listed by analysis groups; themes identified by at least 30% of the group are represented by one figure; themes identified by at least 60% are represented by two figures.

		CF Early (*n* = 16)	CF Late (*n* = 12)	NBS Early (*n* = 11)	NBS Late (*n* = 6)
Communication between NBS programs and CF center	Informal ad hoc meetings	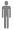	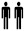 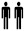	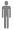	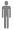
	Formal meetings between CF and NBS programs	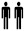	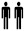	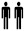	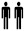
	Minimal communication between CF and NBS programs				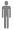
NBS algorithms and laboratory process	IRT cutoffs work well			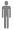	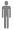
	IRT/DNA algorithm works well	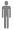			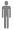
	The *CFTR* panel is not big enough for the population	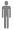			
	The *CFTR* panel is sufficient for population		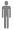		

**Table 2:** Key themes identified listed by analysis groups regarding PCP notification of results to families with an out-of-range CF newborn screen; themes identified by at least 30% of the group are represented by one figure; themes identified by at least 60% of the group are represented by two figures.

		CF Early (*n* = 16)	CF Late (*n* = 12)	NBS Early (*n* = 11)	NBS Late (*n* = 6)
PCP notification of out-of-range results	PCP delayed referral (risk communication)		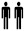		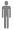
	Miscommunication of results		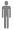		

**Table 3:** Key themes identified listed by analysis groups when interviewees were asked what they wished PCPs better understood about CF; themes identified by at least 30% of the group are represented by one figure; themes identified by at least 60% of the group are represented by two figures.

		CF Early (*n* = 16)	CF Late (*n* = 12)	NBS Early (*n* = 11)	NBS Late (*n* = 6)
What participants wish PCPs better understood about CF NBS	Details of the NBS process (IRT, *CFTR*)		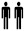	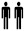	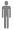
	How to read NBS test results	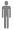		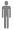	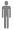
	Communicating risk with the families	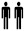	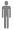		
	Importance of timeliness in CF NBS			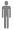	

**Table 4:** Key barriers identified listed by analysis groups regarding the infant or family related to timely sweat testing; themes identified by at least 30% of the group are represented by one figure; themes identified by at least 60% of the group are represented by two figures.

		CF Early (*n* = 16)	CF Late (*n* = 12)	NBS Early (*n* = 11)	NBS Late (*n* = 6)
Barriers related to the famy or infant	Infant age, size, and NICU status	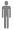		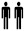	
	Difficulty in reaching family/scheduling sweat test	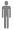	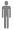	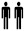	

**Table 5:** Key themes identified listed by analysis groups when participants were asked, “What are the barriers to a timely sweat test for minority groups?”; themes identified by at least 30% of the group are represented by one figure; themes identified by at least 60% are represented by two figures.

		CF Early (*n* = 16)	CF Late (*n* = 12)	NBS Early (*n* = 11)	NBS Late (*n* = 6)
Barriers to a timely sweat test for minority groups	Socioeconomic status	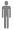	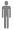	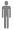	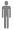
	Geographic distance to sweat testing		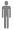		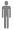
	Transportation	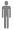		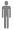	
	Time commitment		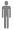		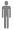
	Missed case due to limited variant panel	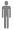		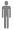	
	Clinical perception of risk		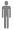		

**Table 6:** Key Themes identified listed by analysis groups regarding the urgency of results called -out to PCPs from the newborn screening program; themes identified by at least 30% of the group are represented by one figure; themes identified by at least 60% of the group are represented by two figures.

		CF Early (*n* = 16)	CF Late (*n* = 12)	NBS Early (*n* = 11)	NBS Late (*n* = 6)
Urgency of call-out for number of variants identified on NBS	More urgent for 2 variants	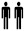	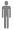	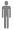	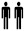
	Less urgent for 1 variant	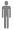	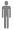	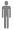	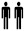
	2 variants: notify PCP, specialists quickly	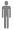	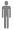	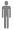	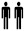
	1 variant: note carrier status and less urgency on notifying and scheduling		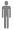	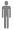	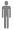
	2 variants: sweat test scheduled quickly	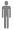			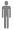
	Treatment initiated (possibly before sweat test) for 2 variants or 1 variant with symptoms.	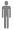		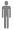	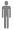

## Data Availability

The data are not publicly available due to the sensitive nature of interview data. The codebook used in this study is available on request from the corresponding author.
